# Gender-based Differential Item Functioning in the Application of the Theory of Planned Behavior for the Study of Entrepreneurial Intentions

**DOI:** 10.3389/fpsyg.2017.00451

**Published:** 2017-03-23

**Authors:** Leonidas A. Zampetakis, Maria Bakatsaki, Charalambos Litos, Konstantinos G. Kafetsios, Vassilis Moustakis

**Affiliations:** ^1^Department of Production Engineering and Management, Technical University of CreteChania, Greece; ^2^Department of Psychology, University of CreteRethymno, Greece

**Keywords:** differential item functioning, gender differences, entrepreneurship, attitudes, intentions, theory of planned behavior

## Abstract

Over the past years the percentage of female entrepreneurs has increased, yet it is still far below of that for males. Although various attempts have been made to explain differences in mens’ and women’s entrepreneurial attitudes and intentions, the extent to which those differences are due to self-report biases has not been yet considered. The present study utilized Differential Item Functioning (DIF) to compare men and women’s reporting on entrepreneurial intentions. DIF occurs in situations where members of different groups show differing probabilities of endorsing an item despite possessing the same level of the ability that the item is intended to measure. Drawing on the theory of planned behavior (TPB), the present study investigated whether constructs such as entrepreneurial attitudes, perceived behavioral control, subjective norms and intention would show gender differences and whether these gender differences could be explained by DIF. Using DIF methods on a dataset of 1800 Greek participants (50.4% female) indicated that differences at the item-level are almost non-existent. Moreover, the differential test functioning (DTF) analysis, which allows assessing the overall impact of DIF effects with all items being taken into account simultaneously, suggested that the effect of DIF across all the items for each scale was negligible. Future research should consider that measurement invariance can be assumed when using TPB constructs for the study of entrepreneurial motivation independent of gender.

## Introduction

Entrepreneurial activity is an important vehicle for value creation and has a significant impact on economic growth, continuous business renewal, and employment (Van Praag and Versloot, 2007). However, although half of the working population are women, and women make up a substantial proportion of those choosing to be entrepreneurs (Minniti et al., 2005), female entrepreneurship significantly lags behind male entrepreneurship (Minniti et al., 2005; Kelley et al., 2013).

According to findings from the Global Entrepreneurship Monitor (GEM) project, males’ rates of entrepreneurial activity range from over three times that of females in some countries, while in others, the male–female ratio of participation is nearly identical (Minniti et al., 2005; Sarri and Trihopoulou, 2005). In nearly all of the 67 economies included in the GEM the rate of men’s venture creation is higher than that of women (Kelley et al., 2013). This is especially true of Greece, which is characterized by higher gender inequality (Sarri and Trihopoulou, 2005).

In the same vein, recent findings from the Global University Entrepreneurial Spirit Students’ Survey project (GUESSS – Sieger et al., 2014; Tognazzo et al., 2016) conducted in 34 countries and in more than 700 universities suggest that 10.7% of all male students strive for an entrepreneurial career path, compared to only 6.6% of all female students. The differences are even larger, 5 years after completion of studies: on average 35.1% of all male students aspire to be entrepreneurs, but only 27.5% of all female students. The aforementioned studies raises questions as to why the rate of men’s venture creation exceeds that of women and what factors explain these differences (Sarri and Trihopoulou, 2005; Piacentini, 2013).

Research has suggested the existence of the gender gap in entrepreneurial orientation and in the motivation, and intention to become an entrepreneur (Mueller and Dato-on, 2013; Schlaegel and Koenig, 2014). The image of the entrepreneur has traditionally been masculinized and rooted in masculine discourse (Ahl, 2006). Moreover, it has been found that for women who work in gender incongruent occupations dominated by men, the experience of discrimination has a negative association with their well-being (Maddox, 2013; Di Marco et al., 2016). Being a member of two traditionally unrelated groups (i.e., being a woman and an entrepreneur) is not an easy task for women (Zampetakis et al., 2016).

Research has drawn on several theoretical perspectives when considering business startup motivation, including innovation theory (Stewart et al., 1999) or social and human capital theory (Langowitz and Minniti, 2007). In recent years Ajzen’s (1991) theory of planned behavior (TPB) is often used as a framework for predicting entrepreneurial motivation (Maes et al., 2014; Schlaegel and Koenig, 2014). According to the TPB, there are three key factors that influence an individual’s intention (INT) to start a business, these being: (i) attitudes toward entrepreneurship (ATT), that is a person’s overall assessment of the advantages and disadvantages of entrepreneurship, (ii) subjective norms (SN), that is a person’s perception of the social pressure from significant others to perform the behavior (i.e., start a business), and (iii) perceived behavioral control (PBC) that is the perceived ease or difficulty of starting a business. The TPB suggests that INT results from positive ATT, positive SN and feelings of control over the creation process.

On average men compared to women have higher INT (Haus et al., 2013). The gender-related differences found in entrepreneurial motivation may be attributable to real and valid differences in constructs used, such as ATT and PBC. According to Maes et al. (2014) women are driven toward entrepreneurship by motives that facilitate a balance in business and personal life, that are less dominant in predicting personal attitude. Moreover, women seem to display lower internal feelings of control than men that are more dominant in predicting PBC.

However, the gender-related differences found in entrepreneurial motivation could also depend on the properties of the instruments being used in research raising issues of construct validity (Bird and Brush, 2002; Jennings and Brush, 2013). What is common in contemporary entrepreneurship research studies is that the often adoption of self-report techniques and structured questionnaires for the assessment of entrepreneurship related variables, such as the ones used in the TPB (Henry et al., 2016). Although scales observe differences in scores between groups, differences may also be due to a characteristic of test items other than the scale attribute. Research on female entrepreneurship has often been criticized for using instruments developed for male entrepreneurs, making it impossible to capture anything differentially feminine while women are more likely to appear inadequate in comparison to men (Stevenson, 1986; Ahl, 2006). These instruments are superimposed on women, and not tested with appropriate methods for measurement equivalence (or Differential item functioning-DIF; Holland and Wainer, 1993), thus missing any potential important differences between the male/female entrepreneurial endeavors.

Differential Item Functioning occurs when a test or a survey item (i.e., a question) functions differently for a reference group (e.g., males) of respondents compared to focal group (e.g., females) respondents, after controlling for the level of the attribute being measured (Millsap, 2012). For example, an item exhibits DIF if the probability of males responding to a specific category differs from females when they both are operating at the same overall level on the construct (Holland and Wainer, 1993; Crane et al., 2006). Awareness of this bias is of particular importance where scale scores are used to investigate gender differences and ensure that derived scores are comparable across groups.

A lack of measurement equivalence at the item level, may lead to spurious mean differences in the observed scores between male and female participants, because one cannot be certain there is a meaningful difference, thereby making mean score differences un-interpretable (Millsap, 2012). Furthermore, the existence of DIF across genders for entrepreneurship-related variables, could lead to scores of questionable meaning and interpretation depending on the gender of the respondent, because DIF suggests that the items do not relate to the construct of interest in the same way. In that situation, scores would not be comparable between males and females; a particular score may have a different meaning for men than it does for women. Taken together, detection of DIF is important as it can influence the psychometric properties of an instrument and mean score comparisons (e.g., Church et al., 2011).

There are several ways in which gender stereotypes, and/or social constructions regarding entrepreneurship and family roles could differentially affect men and women’s responses to entrepreneurship-related constructs. According to gender role theory traditional gender roles prescribe that women’s role should be based around family, while men’s role should be more focused on work (e.g., Gutek et al., 1991). Moreover, entrepreneurship is considered to be a gendered phenomenon (Jennings and Brush, 2013). Because women feel more pressure to have a family centered identity, items such as “A career as entrepreneur is attractive for me”, or “Among various options, I would rather be an entrepreneur” may be interpreted by men and women to indicate differing levels of ATT. Thus, a male respondent and a female respondent with the same moderate level of ATT might answer this item differently. A male respondent might consider his moderate level of ATT as warranting high agreement with these items, since he and the people around him tend to perceive entrepreneurship as a stereotypically masculine endeavor (Jennings and Brush, 2013). A female respondent with the same moderate level of ATT might disagree with this item, since her moderate level might be construed by her and those around her as being too low, as society generally expects women’s identity to reside in the family sphere (i.e., social desirable responding). Socially desirable responding, could influence responses and lead to DIF, as men and women may be uncomfortable providing answers that fall outside of societal expectations.

Similarly, an item on the INT scale such as “Spend time learning about starting a firm” may indicate a different level of INT for men than it would to female respondents. For example, a male respondent and a female respondent with the same high level of INT might respond to such an item differently. The male respondent may endorse strong agreement with the item, since men are generally expected to be more involved in business startup, compared to women.

Nevertheless, the presence of DIF at the item level does not necessarily imply DIF at the scale level (differential test functioning-DTF). Conversely, having no or little DIF at the item level does not imply that the scale as a whole is measurement-invariant (Penfield and Algina, 2006). Research provides evidence that DIF can influence the psychometric properties of test scores (e.g., coefficient alphas, score variances) and depending on its direction, DIF can increase or decrease sum scores (Li and Zumbo, 2009). DIF favoring women might increase women’s scores relative to men’s scores, while DIF favoring men might do the opposite. DTF analyses allow assessing the overall impact of DIF effects with all items being taken into account simultaneously.

Although testing for DIF is a quite common practice in other social science research domains (such as psychology) applications to entrepreneurship related constructs are rare. One notable exception is a study that analyzed the essential dimensions of enterprising personality (Suárez-Álvarez et al., 2014) regarding gender-related DIF. The researchers found that 9 out of the 127 items showed DIF as a function of students’ gender, in constructs such as optimism, innovativeness, self-efficacy, risk taking and stress tolerance. In another study, Maes et al. (2014) used Ajzen’s (1991) TPB as a theoretical framework and analyzed the measurement part of the model, at the indicator level, testing the hypothesis that students’ gender moderates the strength of the relationship between certain indicators and their respective factors. Their analyses indicated important gender differences in the factors that shape entrepreneurial intentions. Finally, entrepreneurial intention is not restricted to students or unemployed people. For example it is plausible that people may have the intention to launch a business while retaining their “day job” for some time (i.e., hybrid entrepreneurs; Raffiee and Feng, 2014).

In summary, although reports of gender-specific differences in constructs used in entrepreneurship research may reflect true distinctions in entrepreneurial intentions between men and women, these same effects may simply be an artifact of gender differences in the linguistics used to describe entrepreneurial phenomena. Given the various mechanisms by which the interpretation of the TPB scales could vary between men and women, the objectives of this study were (1) to test the main antecedents of entrepreneurial behavior (Kautonen et al., 2015) that is, ATT, SN, PBC and INT, using indicators used in previous research for DIF regarding gender and (2) to examine the implications of DIF at the scale level using analyses of DTF.

## Materials and Methods

### Sample and Procedures

Survey data were collected from 1800 individuals from various parts of Greece. The majority of participants (34.1%) were students from various disciplines (e.g., psychology, education, engineering, business and science students). Unemployed participants were 32.5% while 33.4% were employed in the private (17.5%) and the public sector (15.9%).

The study was carried in accordance to the principles expressed in the Declaration of Helsinki and was approved by the authors’ institutional ethics committees. Surveys were administrated to participants through personal contact by the study authors with written informed consent from all participants. A variety of recruitment methods were used, including word of mouth, advertising through social network sites, and course credit. The study was described as examining “Factors affecting career choice and development.” Participants were informed that anonymity was guaranteed and that they had the option to withdraw from the study at any moment. Data collection took place at the beginning of 2016 and lasted approximately 6 months.

In sum, the sample consisted of 1800 participants (50.4% female), the mean sample age was 32.05 years (*SD* = 12.46), range was 18 to 59 years. The majority of respondents (61.8%) had a university/college degree; 433 participants (24.1%) reported that one of their parents owned a full time business most of the time, while they were growing up, 87% reported that they know an entrepreneur in their close environment, and 27% of participants reported that they had some experience from business start-up procedures. The survey instrument contained items representing the theoretical constructs along with demographic data. Items referring to the same construct were positioned in different locations throughout the questionnaire.

### Measurement of Theoretical Constructs

The specific measures used in the analysis, along with sample items of the relevant constructs, are outlined. All the main constructs included in the analysis were assessed with self-report measures based on multi-item scales. The back-translation procedure recommended by Brislin (1980) was followed for the translation of the items into the Greek language.

#### Entrepreneurial Intention (INT)

We assessed participants’ entrepreneurial intent using a scale originally developed by Thompson (2009). This is a reliable and internationally applicable individual entrepreneurial intent scale. It includes ten items, four of which are distracter items that act as red herrings and were not included in scale analyses. Sample items are: “Intend to set up a company in the future,” “I have no plans to launch my own business” (reverse scored). Responses to the six items were made on 7-point Likert-type scales (1 = strongly disagree, 7 = strongly agree). Coefficient alpha for this scale was 0.89.

#### Attitudes Toward Entrepreneurship (ATT)

We assessed ATT using the five item scale from Liñán and Chen (2009). Sample items are: “A career as entrepreneur is attractive for me,” “Among various options, I would rather be an entrepreneur.” Responses to the five items were made on 7-point Likert-type scales (1 = strongly disagree, 7 = strongly agree). Cronbach’s reliability for this scale was 0.88.

#### Subjective Norm (SN)

We assessed SN using the three item scale from Liñán and Chen (2009). Students were asked: “If you decided to create a firm, would people in your close environment approve of that decision?” Items were (a) Your close family, (b) Your friends and (c) your fellow students. Responses to the three items were made on 5-point Likert-type scales (1 = total disapproval, 5 = total approval). Cronbach’s reliability for this scale was 0.80.

#### Perceived Behavioral Control (PBC)

We assessed PBC using five items from the scale of Liñán and Chen (2009). Sample items are: “To start a firm and keep it working would be easy for me,” “I can control the creation process of a new firm.” Responses to the five items were made on 7-point Likert-type scales (1 = strongly disagree, 7 = strongly agree). Cronbach’s reliability for this scale was 0.84.

### Methods of Analyses

First, the fit of the measurement model was examined (that is, the four constructs of the TPB) for the whole sample and separately for men and women. Analysis of Moment Structures (AMOS software, version 7.0) (Arbuckle, 2006) was used. Because the χ^2^ statistic for model fit is highly sensitive to sample size, we employed several statistics to assess model fitness (Shook et al., 2004): (a) Root Mean Square Error Approximation (RMSEA): 0 = an exact fit, <0.05 = a close fit, 0.05–0.08 = a fair fit, 0.08–0.10 = a mediocre fit, and >0.10 = a poor fit (AMOS also computes a 90% confidence interval around RMSEA); (b) Comparative Fit Index (CFI): best if above 0.90; (c) Akaike Information Criterion (AIC). For model comparisons, smaller values in AIC represent a better fit of the model.

Second, DIF analyses were performed. Females served as the focal group with males as the reference group in the gender DIF analyses. The Mantel–Haenszel (MH) χ^2^ procedure, as implemented in the DIFAS (Differential Item Functioning Analysis System – version 5.0) software (Penfield, 2005), was used. The MH statistical procedure consists of comparing the item performance of two groups (reference and focal), whose members were previously matched on the total score of the scale (the matching is done using the observed total test score as a criterion or matching variable). The MH statistic is based on a contingency table analysis. The critical values for this statistic are 3.84 (α = 0.05) and 6.63 (α = 0.01) (Penfield 2013, Unpublished). The results offered by the DIFAS software are displayed in two tables: The first of these shows the DIF statistics, while the second presents the conditional differences in the mean item scores between the reference and focal groups at ten intervals across the matching variable continuum. In the DIF analysis for polytomous items DIFAS software includes several statistics including the MH χ^2^, the Liu-Agresti cumulative common log-odds ratio (L-A LOR), the estimated standard error (SE) of the L-A LOR and the Cox’s Non-centrality Parameter Estimator (COX’S B), with its corresponding SE. The L-A LOR is based on the Haenszel common-odds ratio generalized to polytomous data and represents the log odds ratio of one group selecting a response option compared with the other group when the level of the overall measured construct is the same (Penfield 2013, Unpublished). Positive values indicate DIF in favor of the reference group, and negative values indicate DIF in favor of the focal group. The standardized Liu-Agresti Cumulative Common Log-Odds Ratios (LOR Z) was also used. A value greater than 2.0 or less than -2.0 may be considered evidence of the presence of DIF (Penfield and Algina, 2003). Finally, Cox’s B is similar to the MH statistic except that it uses the hypergeometric mean. It is distributed similarly to L-A LOR that is, positive values indicate DIF in favor of the reference group, and negative values indicate DIF in favor of the focal groups. The size of the DIF was interpreted using a widely accepted classifying system whereby DIF in polytomous items is considered negligible if L-A LOR < 0.43, moderate if between 0.43 and 0.64, and large if >0.64 (Penfield, 2007).

Third, DTF analysis was conducted to examine measurement invariance directly at the scale level and was analyzed using the ν^2^ statistic in DIFAS (version 5.0) (Penfield, 2005, 2013 Unpublished). The ν^2^ statistic allows quantifying the overall DIF effect across the items of a scale (Penfield and Algina, 2006). A scale with a DIF effect variance of ν^2^ below 0.07 can be classified as having small DTF, whereas DTF would be considered medium for 0.07 ≤ ν^2^ ≤ 0.14 and large for ν^2^ > 0.14 (Penfield and Algina, 2006; Penfield 2013, Unpublished). To examine whether differential functioning of the items influenced gender differences on the TPB scales, we computed Cohen’s *d* for gender differences (Cohen, 1988) for each scale. First, Cohen’s *d* was computed using all items, next items with large level of DIF were removed and lastly items with moderate a large levels of DIF were removed.

## Results

### Descriptive Summary and Correlations

We present means, standard deviations and correlations across the four variables of the TPB, for the entire sample and separately for men and women participating in the study, in **Tables 1**–**3**.

**Table 1:** Descriptive statistics and inter-correlations for the total sample (*N* = 1800).

	*M*	*SD*	1	2	3	4	5	6
1. Gender ^a^	1.50	0.50	–					
2. Age	32.05	12.46	0.05	–				
3. ATT	4.29	1.44	-0.13**	-0.01	(0.88)			
4. PBC	2.81	1.24	-0.17**	0.07**	0.45**	(0.83)		
5. SN	4.66	1.42	-0.05*	-0.18**	0.34**	0.29**	(0.80)	
6. INT	2.63	1.40	-0.18*	-0.03	0.54**	0.68**	0.26**	(0.89)


*^a^ Gender is coded: 1 = male 2 = female; Cronbach’s alpha reliabilities are in parenthesis. ^∗^*p < 0.05 (two tailed)*,^∗∗^*p < 0.01 (two tailed).**

**Table 2:** Descriptive statistics and inter-correlations for men in the sample (*N* = 892).

	*M*	*SD*	1	2	3	4	5
1. Age	31.98	12.66	–				
2. ATT	4.49	1.41	0.07	(0.87)			
3. PBC	3.03	1.27	0.09^∗∗^	0.45^∗∗^	(0.84)		
4. SN	4.73	1.43	–0.18^∗∗^	0.33^∗∗^	0.26^∗∗^	(0.80)	
5. INT	2.89	1.46	0.02	0.54^∗∗^	0.67^∗∗^	0.26^∗∗^	(0.88)


*Cronbach’s alpha reliabilities are in parenthesis. ^∗^*p < 0.05 (two tailed)*,^∗∗^*p < 0.01 (two tailed).**

**Table 3:** Descriptive statistics and inter-correlations for women in the sample (*N* = 908).

	*M*	*SD*	1	2	3	4	5
1. Age	32.11	12.27	–				
2. ATT	4.11	1.45	0.03	(0.89)			
3. PBC	2.60	1.17	0.06	0.43^∗∗^	(0.86)		
4. SN	4.60	1.40	-0.17^∗∗^	0.35^∗∗^	0.31^∗∗^	(0.81)	
5. INT	2.38	1.28	-0.06	0.53^∗∗^	0.67^∗∗^	0.25^∗∗^	(0.88)


*Cronbach’s alpha reliabilities are in parenthesis. ^∗∗^*p < 0.01 (two tailed).**

Results of independent *t*-tests, suggested that men scored higher compared to women in ATT [Men: *M*_ATT_ = 4.49; Women: *M*_ATT_ = 4.11; *t* (1798) = 5.572, *p* < 0.001], PBC [Men: *M*_ATT_ = 3.03; Women: *M*_ATT_ = 2.60; *t* (1780) = 7.432, *p* < 0.001], SN [Men: *M*_ATT_ = 4.73; Women: *M*_ATT_ = 4.60; *t* (1798) = 2.117, *p* = 0.034] and INT [Men: *M*_ATT_ = 2.89; Women: *M*_ATT_ = 2.38; *t* (1762.19) = 7.953, *p* < 0.001] (**Tables 2**, **3**). These results are in line with previous research suggesting significant gender differences in terms of perceived feasibility (expressed as PBC), perceived desirability (expressed as ATT) and INT (Kolvereid, 1996; Dabic et al., 2012; Sieger et al., 2014). Moreover, results from one way ANOVA analyses suggested that employees working in the private sector and unemployed had higher INT compared to the other two groups of participants. We have found no statistically significant differences between students and participants working in the public sector in terms of ATT, PBC and INT; students scored higher to SN [*t* (475.77) = -5.897, *p* < 0.001].

### Confirmatory Factor Analyses

Results from the confirmatory factor analysis (CFA) of the measurement model for the whole sample, suggested an adequate fit to the data: χ^2^ (146, *N* = 1800) = 1796.39, *p* = 0.000; RMSEA = 0.079 (90% CI: 0.075-0.081); CFI = 0.916; AIC = 1922.39. All factor loadings are significant at the 0.001 level. To further assess discriminant validity of the constructs, we compared the measurement model with a model that constrained the correlations among the constructs to be equal and examined the change in chi-square (χ^2^). A model comparison between the unconstrained measurement model and a model that constrained the correlations among the constructs to be equal produces a significant difference in χ^2^, suggesting the presence of discriminant validity among the selected constructs (Δχ^2^ = 634.97, Δ*df* = 5, *p* < 0.001). The fit indices indicated also an adequate model fit for women [χ^2^ (146, *N* = 908) = 1048.13, *p* = 0.000; RMSEA = 0.083 (90% CI: 0.076-0.087); CFI = 0.911; AIC = 1174.13] and men participants [χ^2^ (151, *N* = 892) = 999.03, *p* = 0.000; RMSEA = 0.081 (90% CI: 0.076-0.085); CFI = 0.908; AIC = 1125.03].

### Differential Item Functioning (DIF)

In **Table 4** we present the Mantel χ^2^, L-A LOR, LOR Z and COX’S *B*-values for all the items in the four constructs. One item in the ATT scale: Item 4 – “Being an entrepreneur would entail great satisfactions for me,” exhibited a statistically significant but negligible DIF based on the L-A LOR criteria outlined above (Mantel χ^2^ = 2.871, *p* < 0.10) (Penfield, 2007). No DIF was found for the PBC and SN scale. Finally one item in the INT scale: Item 6- “Spend time learning about starting a firm” exhibited a statistically significant but negligible DIF (Mantel χ^2^ = 4.566, *p* < 0.05). The negative L-ALOR of the item (4) in the ATT scale indicates DIF favoring the focal group (women), i.e., for the same level of construct easier to endorse for women. The positive L-ALOR of the item (6) in the INT scale indicates DIF favoring men.

**Table 4:** Results of the differential item functioning (DIF) analyses.

Constructs	Item content	Mantel χ^2^	L-A LOR *(SE)*	LOR *(Z)*	COX’S B *(SE)*
1. ATT	1. Being an entrepreneur implies more advantages than disadvantages to me	0.806	0.082 (0.092)	0.891	0.042 (0.046)
	2. A career as entrepreneur is attractive for me	0.433	-0.064 (0.097)	-0.660	-0.035 (0.053)
	3. If I had the opportunity and resources, I’d like to start a firm	1.447	0.116 (0.097)	1.196	0.062 (0.052)
	4. Being an entrepreneur would entail great satisfactions for me	**2.871**^∗^	-0.167 (0.099)	-1.687	-0.1 (0.059)
	5. Among various options, I would rather be an entrepreneur	0.007	-0.008 (0.097)	0.082	-0.005 (0.055)
2. PBC	1. To start a firm and keep it working would be easy for me	2.198	0.146 (0.098)	1.49	0.073 (0.0494)
	2. I am prepared to start a viable firm	0.075	0.029 (0.11)	0.264	0.017 (0.0605)
	3. I can control the creation process of a new firm	2.342	-0.15 (0.098)	-1.531	-0.082 (0.0536)
	4. I know the necessary practical details to start a firm	0.306	0.058 (0.103)	0.563	0.025 (0.046)
	5. If I tried to start a firm, I would have a high probability of succeeding	0.709	-0.079 (0.095)	-0.832	-0.039 (0.0468)
3. SN	1. Your close family	0.032	0.018 (0.099)	0.182	0.009 (0.049)
	2. Your friends	1.896	0.144 (0.104)	1.385	0.09 (0.0655)
	3. Your colleagues	1.568	-0.124 (0.1)	-1.24	-0.064 (0.051)
4. INT	1. Intend to set up a company in the future	0.439	0.064 (0.096)	0.667	0.025 (0.038)
	2. Never search for business start-up opportunities	0.053	-0.024 (0.105)	-0.229	-0.011 (0.047)
	3. Are saving money to start a business	2.144	-0.162 (0.109)	-1.486	-0.073 (0.051)
	4. Do not read books on how to set up a firm	0.013	-0.014 (0.118)	-0.119	-0.006 (0.053)
	5. Have no plans to launch your own business	1.19	-0.116 (0.107)	-1.084	-0.058 (0.054)
	6. Spend time learning about starting a firm	**4.566** ^∗∗^	0.232 (0.111)	2.09	0.115 (0.054)


*^∗^*p* < 0.10; ^∗∗^*p* < 0.05; Negative L-A LOR values indicate DIF favoring the focal group (women), i.e., for the same level of construct easier to endorse for the focal group. Conversely, positive L-A LOR values indicate DIF favoring men. Classification for L-A LOR: DIF is considered negligible if L-A LOR < 0.43, moderate if between 0.43 and 0.64, and large if >0.64. Results meeting important criteria for DIF are marked in bold.*

### Differential Test functioning (DTF)

We present the ν^2^ coefficients for the four TPB contsructs in **Table 5**. Based on criteria for assessing the size of DTF (Penfield and Algina, 2006), the DTFs were deemed not to warrant concern (all ν^2^ coefficients, were below 0.07).

**Table 5:** Results of the differential test functioning (DTF) analyses.

Scale	ν^2^ (*SE*)
1. ATT	0.001 (0.007)
2. PBC	0.002 (0.006)
3. SN	0.003 (0.01)
4. INT	0.005 (0.009)


## Discussion

The primary goal of this study was to investigate the validity and meaningfulness of the main antecedents of entrepreneurial behavior (Kautonen et al., 2015) that is, ATT, SN, PBC and entrepreneurial intentions across gender. Such comparisons have potential theoretical importance in increasing researchers’ understanding of the interplay between gender and entrepreneurial motivation and improve the participation rate of women in entrepreneurial activities. We focused on one important prerequisite for such comparisons, measurement invariance. To our knowledge, this is the first examination of gender-based DIF in entrepreneurship-related constructs.

Specifically, this study addressed DIF in the constructs that constitute the TPB, a widely used theoretical framework for the study of entrepreneurial motivation. Our results suggest that there are overall differences in mean scores for men and women in the TPB dimensions, yet the DIF analysis indicated that differences at the item-level are almost non-existent. Men outperformed women in ATT, PBC, SN and INT. These results are in agreement with previous studies concerning gender differences in entrepreneurial attitudes and intentions (Haus et al., 2013; Tognazzo et al., 2016). Moreover, the DTF analysis suggested that the effect of DIF across all the items for each scale was negligible.

The study contributes to previous research that uses the TPB model to study entrepreneurial intentions (Maes et al., 2014; Schlaegel and Koenig, 2014). Our results suggest that after controlling for the underlying TPB construct, the response to an item is not related to whether the respondent is male or female. Thus, the TPB constructs appear to function equivalently for men and women at the item level. Furthermore, our DTF analyses for each TPB construct, where we assessed the overall impact of DIF effects with all items being taken into account simultaneously, suggested that the scales of the TPB as whole are measurement invariant. These findings provide evidence that the constructs used in the present research provide valid comparisons between male and female respondents.

Our findings suggest that actually women tend to demonstrate lower entrepreneurial intentions compared to men (at least in a country such as Greece) and this gender-related difference is not dependent on the properties of the instrument being used. This opens the road for researchers to examine other theoretical variables that influence the lower entrepreneurial intention of women, For example Zampetakis et al. (2016) proposed that gender identity, that is the extent to which people incorporate gender roles into their self-concepts, is a promising construct for the study of gender differences in intentions related to entrepreneurship.

Although our study sheds some light on measurement invariance of the TPB constructs applied to entrepreneurship across gender, it has several limitations that further research can seek to address. First, our study design is cross sectional, where we did not measure actual business startup, but only respondents’ intent to start a business. As such, one could consider our INT construct as general attitude to become an entrepreneur. Although our CFA results suggest that ATT and INT are two separate factors, future research could employ longitudinal designs, including actual business startup, in order to validate the INT construct.

Second, our study was limited to a sample of Greek participants. To extend the generalizability of our results, we encourage scholars in this area to examine our proposed model with different samples across different countries. Second, we applied non-parametric DIF detection methods. Non-parametric methods make fewer assumptions concerning the distribution of the latent trait in the population, but have the disadvantage that they rely on an observed score as the matching variable. This suggests that if our measurement contain widespread bias, it is possible that some bias within the measurement was not detected. Future research could use parametric DIF estimates in the framework of item response theory (IRT); IRT-based methods use a latent variable modeling approach.

Third, our analyses were based on manifest grouping variables such as gender, where DIF and DTF results depend on the contrasting group. Future research could benefit from the use of latent DIF detection approaches that relies on the use of mixture IRT models, that is, a combination of IRT and latent class models (Benítez et al., 2016). The use of mixture IRT models to detect DIF differentiates groups based on an unknown latent grouping variable that is not specified *a priori* but is determined by the results from the model parameter estimation.

One last consideration concerns the social and economic context in which the study took place. The recent global economic crisis with its peak in 2008 resulted in shocking changes for the labor market: in many countries workers lost their jobs, the work hours shortened while wage earnings declined (Pines et al., 2010; Giorgi et al., 2015). Greece is facing severe economic challenges in recent years. The economic crisis is an important stressor with negative effects on the health of workers and especially women (Mucci et al., 2016). According to Drydakis (2015) during the Greek economic crisis, women were more negatively affected by unemployment in terms of their physical and mental health in comparison to men. Higher stress regarding employment status may exacerbate gender roles and may have further influenced relative cross-gender differences in entrepreneurial intentions and attitudes.

## Conclusion

The present study examined DIF analysis in constructs of the TPB, a theoretical framework that is often used for describing entrepreneurial intention. DIF analysis indicated that differences at the item and scale level are almost non-existent between male and female participants. However, DIF results may not generalize across inventories, especially when they have different theoretical frameworks. As such we believe that DIF should be conducted in constructs used in different theoretical frameworks of entrepreneurship research as testing for DIF enables developers to determine whether the constructs behave differently for women and men. In our opinion, DIF should be a prerequisite of meaningful group comparisons across male and female respondents, for the study of entrepreneurship related phenomena.

## Author Contributions

LZ conceptualized the study; LZ, KK, and VM designed the study; LZ, MB, CL, and KK performed research; LZ analyzed data; LZ and KK wrote the manuscript; MB and CL read and corrected the manuscript; VM and KK supervised the entire project. All authors listed, have made substantial, direct and intellectual contribution to the work, and approved it for publication.

## Conflict of Interest Statement

The authors declare that the research was conducted in the absence of any commercial or financial relationships that could be construed as a potential conflict of interest.

## References

[B1] Ahl H. (2006). Why research on women entrepreneurs needs new directions. *Entrep. Theory Pract.* 30 595–621. 10.1111/j.1540-6520.2006.00138.x

[B2] Ajzen I. (1991). The theory of planned behavior. *Organ. Behav. Hum. Decis. Process.* 50 179–211. 10.1016/0749-5978(91)90020-T

[B3] Arbuckle J. L. (2006). *AMOS 7.0. User Guide.* Chicago, IL: Small Waters Corporation.

[B4] Benítez I. Padilla J. L. Hidalgo Montesinos M. D. Sireci S. G. (2016). Using mixed methods to interpret differential item functioning. *Appl. Meas. Educ.* 29 1–16. 10.1080/08957347.2015.1102915

[B5] Bird B. Brush C. (2002). A gendered perspective on organizational creation. *Entrep. Theory Pract.* 26 41–66.

[B6] Brislin R. W. (1980). “Translation and content analysis of oral and written material,” in *Handbook of Cross Cultural Psychology* Vol. 2 eds Triandis H. C. Berry J. W. (Boston, MA: Allyn & Bacon), 398–444.

[B7] Church A. T. Alvarez J. M. Mai N. T. French B. F. Katigbak M. S. Ortiz F. A. (2011). Are cross-cultural comparisons of personality profiles meaningful? Differential item and facet functioning in the Revised NEO Personality Inventory. *J. Pers. Soc. Psychol.* 101 1068–1089. 10.1037/a0025290 21910552

[B8] Cohen J. (1988). *Statistical Power Analysis for the Behavioral Sciences*, 2nd Edn Hillsdale, NJ: Lawrence Earlbaum Associates.

[B9] Crane P. K. Gibbons L. E. Jolley L. van Belle G. (2006). Differential item functioning analysis with ordinal logistic regression techniques: DIFdetect and difwithpar. *Med. Care* 44 S115–S123. 10.1097/01.mlr.0000245183.28384.ed 17060818

[B10] Dabic M. Daim T. Bayraktaroglu E. Novak I. Basic M. (2012). Exploring gender differences in attitudes of university students towards entrepreneurship: an international survey. *Int. J. Gend. Entrep.* 4 316–336. 10.1108/17566261211264172

[B11] Di Marco D. López-Cabrera R. Arenas A. Giorgi G. Arcangeli G. Mucci N. (2016). Approaching the discriminatory work environment as stressor: the protective role of job satisfaction on health. *Front. Psychol.* 7:1313 10.3389/fpsyg.2016.01313 PMC5003878 27625625

[B12] Drydakis N. (2015). The effect of unemployment on self-reported health and mental health in Greece from 2008 to 2013: a longitudinal study before and during the financial crisis. *Soc. Sci. Med.* 128 43–51. 10.1016/j.socscimed.2014.12.025 25589031

[B13] Giorgi G. Arcangeli G. Mucci N. Cupelli V. (2015). Economic stress in the workplace: the impact of fear of the crisis on mental health. *Work* 5 135–142. 10.3233/WOR-141844 24594539

[B14] Gutek B. A. Searle S. Klepa L. (1991). Rational versus gender role explanations for work-family conflict. *J. Appl. Psychol.* 76 560–568. 10.1037/0021-9010.76.4.560

[B15] Haus I. Steinmetz H. Isidor R. Kabst R. (2013). Gender effects on entrepreneurial intention: a meta-analytical structural equation model. *Int. J. Gend. Entrep.* 5 130–156. 10.1108/17566261311328828

[B16] Henry C. Foss L. Ahl H. (2016). Gender and entrepreneurship research: a review of methodological approaches. *Int. Small Bus. J.* 34 217–341. 10.1177/0266242614549779

[B17] Holland P. W. Wainer H. (1993). *Differential Item Functioning.* Hillsdale, NJ: Erlbaum.

[B18] Jennings J. E. Brush C. G. (2013). Research on women entrepreneurs: challenges to (and from) the broader entrepreneurship literature? *Acad. Manage. Ann.* 7 661–713. 10.1080/19416520.2013.782190

[B19] Kautonen T. Van Gelderen M. Fink M. (2015). Robustness of the theory of planned behavior in predicting entrepreneurial intentions and actions. *Entrep. Theory Pract.* 39 655–674. 10.1111/etap.12056

[B20] Kelley D. Brush C. Greene P. Litovsky Y. (2013). *Global Entrepreneurship Monitor 2012 Women’s Report.* Wellesley, MA: Babson College.

[B21] Kolvereid L. (1996). Prediction of employment status choice intentions. *Entrep. Theory Pract.* 21 47–57.

[B22] Langowitz N. Minniti M. (2007). The entrepreneurial propensity of women. *Entrep.Theory Pract.* 31 341–364. 10.1111/j.1540-6520.2007.00177.x

[B23] Li Z. Zumbo B. D. (2009). Impact of differential item functioning on subsequent statistical conclusions based on observed test score data. *Psicologica* 30 343–370.

[B24] Liñán F. Chen Y. W. (2009). Development and cross-cultural application of a specific instrument to measure entrepreneurial intentions. *Entrep. Theory Pract.* 33 593–617. 10.1111/j.1540-6520.2009.00318.x

[B25] Maddox T. (2013). Professional women’s well-being: the role of discrimination and occupational characteristics. *Women Health* 53 706–729. 10.1080/03630242.2013.822455 24093451 PMC3806220

[B26] Maes J. Leroy H. Sels L. (2014). Gender differences in entrepreneurial intentions: a TPB multi-group analysis at factor and indicator level. *Eur. Manage. J.* 32 784–794. 10.1016/j.emj.2014.01.001

[B27] Millsap R. E. (2012). *Statistical Approaches to Measurement Invariance.* New York, NY: Routledge- Taylor & Francis Group.

[B28] Minniti M. Arenius P. Langowitz N. (2005). *Global Entrepreneurship Monitor 2004 Report on Women and Entrepreneurship.* Babson Park, MA: The Center for Women’s Leadership at Babson College.

[B29] Mucci N. Giorgi G. Roncaioli M. Perez J. F. Arcangeli G. (2016). The correlation between stress and economic crisis: a systematic review. *Neuropsychiatr. Dis. Treat.* 12 983–993. 10.2147/NDT.S98525 27143898 PMC4844458

[B30] Mueller S. L. Dato-on M. C. (2013). A cross cultural study of gender-role orientation and entrepreneurial self-efficacy. *Int. Entrep. Manage. J.* 9 1–20. 10.1007/s11365-011-0187-y

[B31] Penfield R. D. (2005). DIFAS: differential item functioning analysis system. *Appl. Psychol. Meas.* 29 150–151. 10.1177/0146621603260686

[B32] Penfield R. D. (2007). An approach for categorizing DIF in polytomous items. *Appl. Meas. Educ.* 20 335–355. 10.1080/08957340701431435

[B33] Penfield R. D. Algina J. (2003). Applying the Liu-Agresti estimator of the cumulative common odds ratio to DIF detection in polytomous items. *J. Educ. Meas.* 40 353–370. 10.1111/j.1745-3984.2003.tb01151.x

[B34] Penfield R. D. Algina J. (2006). A generalized DIF effect variance estimator for measuring unsigned differential test functioning in mixed format tests. *J. Educ. Meas.* 43 295–312. 10.1111/j.1745-3984.2006.00018.x

[B35] Piacentini M. (2013). “Women entrepreneurs in the OECD: key evidence and policy challenges,” in *Paper Presented at the OECD Social, Employment and Migration Working Papers, Paris*.

[B36] Pines M. A. Lerner M. Schwartz D. (2010). Gender differences in entrepreneurship: equality, diversity and inclusion in times of global crisis. *Equal. Divers. Incl.* 29 186–198. 10.1108/02610151011024493

[B37] Raffiee J. Feng J. (2014). Should I quit my day job? A hybrid path to entrepreneurship. *Acad. Manage. J.* 57 936–963. 10.5465/amj.2012.0522

[B38] Sarri K. Trihopoulou A. (2005). Female entrepreneurs’ personal characteristics and motivation: a review of the Greek situation. *Women Manage. Rev.* 20 24–36. 10.1108/09649420510579559

[B39] Schlaegel C. Koenig M. (2014). Determinants of entrepreneurial intent: a meta-analytic test and integration of competing models. *Entrep. Theory Pract.* 38 291–332. 10.1111/etap.12087

[B40] Shook C. L. Ketchen D. J. J. Hult G. T. M. Kacmar K. M. (2004). An assessment of the use of structural equation models in strategic management research. *Strateg. Manage. J.* 25 397–404. 10.1097/HMR.0b013e3181d5b0f5

[B41] Sieger P. Fueglistaller U. Zellweger T. (2014). *Student entrepreneurship across the Globe: a look at intentions and activities. International Report of the GUESSS Project 2013/2014.* St.Gallen: Swiss Research Institute of Small Business and Entrepreneurship.

[B42] Stevenson L. (1986). Against all odds: the entrepreneurship of women. *J. Small Bus. Manage.* 24 30–36.

[B43] Stewart W. H. Watson W. E. Carland J. C. Carland J. W. (1999). A proclivity for entrepreneurship: a comparison of entrepreneurs, small business owners, and corporate managers. *J. Bus. Ventur.* 14 189–214. 10.1016/S0883-9026(97)00070-0

[B44] Suárez-Álvarez J. Pedrosa I. García-Cueto E. Muñiz J. (2014). Screening enterprising personality in youth: an empirical model. *Span. J. Psychol.* 17 E60 10.1017/sjp.2014.61 26055899

[B45] Thompson E. R. (2009). Individual entrepreneurial intent: construct clarification and development of an internationally reliable metric. *Entrep. Theory Pract.* 33 669–694. 10.1111/j.1540-6520.2009.00321.x

[B46] Tognazzo A. Gubitta P. Gianecchini M. Gianecchini M. (2016). “My old and my new family” - the impact of family relationships on students’ entrepreneurial intentions: an Italian study. *Int. Rev. Entrep.* 14 447–468.

[B47] Van Praag C. M. Versloot P. (2007). What is the value of entrepreneurship? A review of recent research. *Small Bus. Econ.* 29 351–382. 10.1007/s11187-007-9074-x

[B48] Zampetakis L. A. Bakatsaki M. Kafetsios K. Moustakis V. (2016). Sex differences in entrepreneurs’ business growth intentions: an identity approach. *J. Innov. Entrep.* 5 29 10.1186/s13731-016-0057-5

